# Application of deep learning and feature selection technique on external root resorption identification on CBCT images

**DOI:** 10.1186/s12903-024-03910-w

**Published:** 2024-02-19

**Authors:** Nor Hidayah Reduwan, Azwatee Abdul Aziz, Roziana Mohd Razi, Erma Rahayu Mohd Faizal Abdullah, Seyed Matin Mazloom Nezhad, Meghna Gohain, Norliza Ibrahim

**Affiliations:** 1 Department of Oral and Maxillofacial Clinical Sciences, Faculty of Dentistry, Universiti Malaya, Kuala Lumpur, 50603 Malaysia; 2 Department of Restorative Dentistry, Faculty of Dentistry, Universiti Malaya, Kuala Lumpur, 50603 Malaysia; 3 Department of Pediatric Dentistry and Orthodontic, Faculty of Dentistry, Universiti Malaya, Kuala Lumpur, 50603 Malaysia; 4 Department of Artificial Intelligence, Faculty of Computer Science and Information Technology, Universiti Malaya, Kuala Lumpur, 50603 Malaysia; 5 Centre of Oral and Maxillofacial Diagnostic and Medicine Studies, Faculty of Dentistry, University Teknologi MARA, Sungai Buloh, 47000 Malaysia

**Keywords:** External root resorption, Cone beam computed tomography, Artificial intelligence, Deep learning, Feature selection technique, Classification

## Abstract

**Background:**

Artificial intelligence has been proven to improve the identification of various maxillofacial lesions. The aim of the current study is two-fold: to assess the performance of four deep learning models (DLM) in external root resorption (ERR) identification and to assess the effect of combining feature selection technique (FST) with DLM on their ability in ERR identification.

**Methods:**

External root resorption was simulated on 88 extracted premolar teeth using tungsten bur in different depths (0.5 mm, 1 mm, and 2 mm). All teeth were scanned using a Cone beam CT (Carestream Dental, Atlanta, GA). Afterward, a training (70%), validation (10%), and test (20%) dataset were established. The performance of four DLMs including Random Forest (RF) + Visual Geometry Group 16 (VGG), RF + EfficienNetB4 (EFNET), Support Vector Machine (SVM) + VGG, and SVM + EFNET) and four hybrid models (DLM + FST: (i) FS + RF + VGG, (ii) FS + RF + EFNET, (iii) FS + SVM + VGG and (iv) FS + SVM + EFNET) was compared. Five performance parameters were assessed: classification accuracy, F1-score, precision, specificity, and error rate. FST algorithms (Boruta and Recursive Feature Selection) were combined with the DLMs to assess their performance.

**Results:**

RF + VGG exhibited the highest performance in identifying ERR, followed by the other tested models. Similarly, FST combined with RF + VGG outperformed other models with classification accuracy, F1-score, precision, and specificity of 81.9%, weighted accuracy of 83%, and area under the curve (AUC) of 96%. Kruskal Wallis test revealed a significant difference (*p* = 0.008) in the prediction accuracy among the eight DLMs.

**Conclusion:**

In general, all DLMs have similar performance on ERR identification. However, the performance can be improved by combining FST with DLMs.

**Supplementary Information:**

The online version contains supplementary material available at 10.1186/s12903-024-03910-w.

## Introduction

 Early detection of ERR is crucial as it may lead to progressive, irreversible damage and tooth loss in severe cases [1, 2]. ERR is commonly revealed incidentally during radiographic examination though the prevalence has been reported as high as 28.8% [3, 4]. The periapical radiograph is one of the radiographic examinations commonly used to identify ERR. Although it has a high resolution, this image has several limitations, such as superimposition of two-dimensional image that may underestimate the true extent of ERR [5, 6]. It has been reported that CBCT is superior to intra-oral periapical radiographs in detecting ERR because it permits three-dimensional evaluation [6–10]. However, assessment of ERR on CBCT can be influenced by observer performance and viewing condition. A computer-aided tool may improve the identification and reduce the time in identifying pathologies such as ERR [11].

In dentistry, machine learning (ML), a subfield of artificial intelligence (AI) based tools, has been developed to automate the identification of oral and maxillofacial pathologies such as ERR [12]. Random Forest (RF) and Support Vector Machine (SVM) classifiers were the high-performing ML algorithms commonly utilized for image classification tasks in dentistry [13–15]. The use of multilayer convolutional neural networks (CNN) contributes to deep learning (DL) methods that can learn image features and perform classification tasks [16–18]. However, CNN requires high computational costs and needs to adapt a considerable number of parameters [19]. To address this issue, several pre-trained models have been established with pre-defined network architectures. To overcome the issue of overfitting due to limited sample data for deep learning training, a transfer learning with CNN had been recommended for small sample size studies [20]. Transfer learning model based on Visual Geometry Group with 16-layer (VGG16) and EfficientNetB4 has been reported to achieve excellent performance on several image classification tasks [19, 21, 22]. The ensemble of pre-trained architectures such as VGG16 and EfficientNetB4 with machine learning algorithms (RF and SVM) have resulted in high performance of classification tasks [19].

The development of machine learning models that incorporate medical diagnostic images for disease classification has encountered significant challenges resulting from the complex and large number of features present in these images [23]. To address this challenge, a process known as the feature selection technique (FST) was introduced. The process is specifically designed to extract the most relevant and significant subset from the original set of features [24]. Feature selection technique has been implemented to classify carious cavities with a reported high accuracy of 96% [25]. Another study on FST for breast cancer classification found that using conventional FST improved classification accuracy by 51% [26]. A novel FST wrapper method (Boruta algorithm) has recently been implemented to improve the performance of RF classifiers in classification models [27]. Additionally, to improve the performance of SVM classifiers, the RFE algorithm was widely used as a feature selection method [28, 29].

The current study utilised an innovative approach that combines transfer learning using VGG16 and EFNetB4 architectures. This novel methodology incorporates the integration of Support Vector Machine (SVM) and Random Forest (RF) classifiers to improve the accuracy of ERR classification. In this study, four deep learning models, which consist of a hybrid between ML algorithms (RF and SVM) with pre-trained DL architectures (VGG16 and EfficientnetB4) were developed for the identification and classification of ERR. Feature selection methods were implemented on all models to optimize the classification performance of ERR resulting in four additional optimised models. Therefore, this study aims to (1) evaluate the accuracy of deep learning models (DLMs) in ERR identification, (2) assess the effect of FST on DLM performance.

## Materials and methods

### Study protocol

**Fig. 1:**
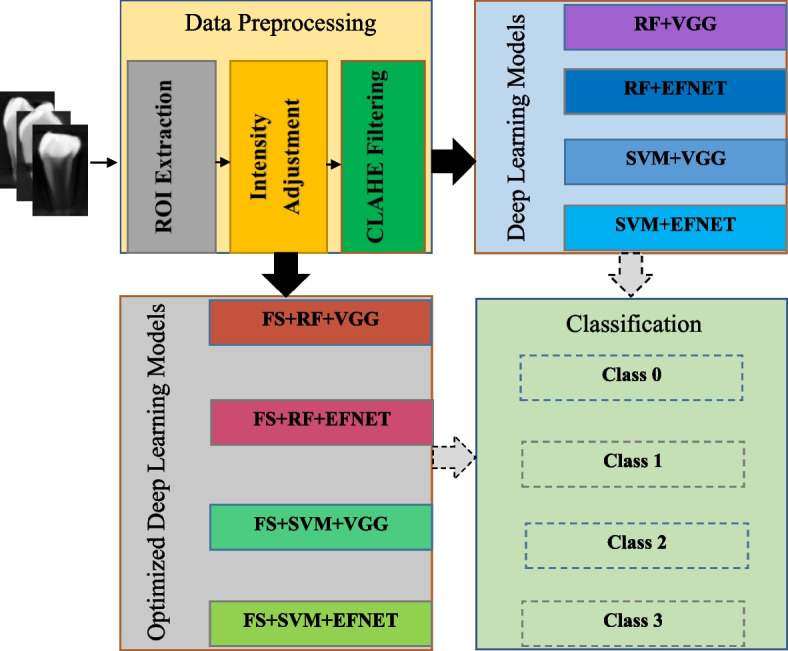
Proposed model block diagram

This study assessed the effect of feature selection technique (FST) on DLM performance in classifying ERR lesions. In the first stage of this study, image preprocessing was performed using Contrast-Limited Adaptive Histogram Equalization (CLAHE) filter. Then, image classification analysis was conducted using two pretrained deep convolutional neural networks (CNN), namely, EfficientNetB4 [30] and VGG16. These two deep CNNs were ensemble with two machine learning classifiers; Random Forest (RF) and Support Vector Machine (SVM) to perform ERR classification. As a result, four DLMs were developed; RF with VGG16 (RF + VGG), RF with EfficientnetB4 (RF + EFNET), SVM with VGG16 (SVM + VGG) and SVM with EfficientnetB4 (SVM + EFNET) in the first stage. In the second stage, a feature selection algorithm (Boruta and RFE) was employed to generate four new optimized DLMs (FS + RF + VGG, FS + RF + EFNET, FS + SVM + VGG and FS + SVM + EFNET) [30]. The block diagram of the proposed model as discussed in the study protocol is given in Fig. 1. The Institutional Review Board of the Medical Ethics Committee Faculty of Dentistry University of Malaya (DF RD2030/0139 (L)) has reviewed and approved this study protocol.

### Dataset

A total of 88 extracted premolars were collected from the Faculty of Dentistry, University of Malaya. The inclusion criteria set for this study were absence of root destruction, complete root formation, absence of caries or abrasions in the cervical region, and no endodontic treatment [31]. Tungsten burrs of various sizes (0.5 mm, 1.0 mm, and 2.0 mm) were used to simulate different depths ERR on each tooth. All teeth were scanned with a CBCT machine (CS 9000 CBCT, Carestream Dental, Atlanta, GA). The acquisition settings were 65 kVp, 5 mA, 10.8 S 5 × 3 cm F.O.V., 0.076 mm isotropic voxel size. In total, 2125 2D slices of CBCT images were obtained. All CBCT datasets were converted to Digital Imaging and Communication in Medicine (DICOM) format. The sample size was calculated based on a previous comparable study [26] by *a priori* power analysis in G*power 3.1.9.7, assuming an independent *t*-test dataset with a power of 80% and significance of 5%.

### Ground truth labelling

**Fig. 2:**
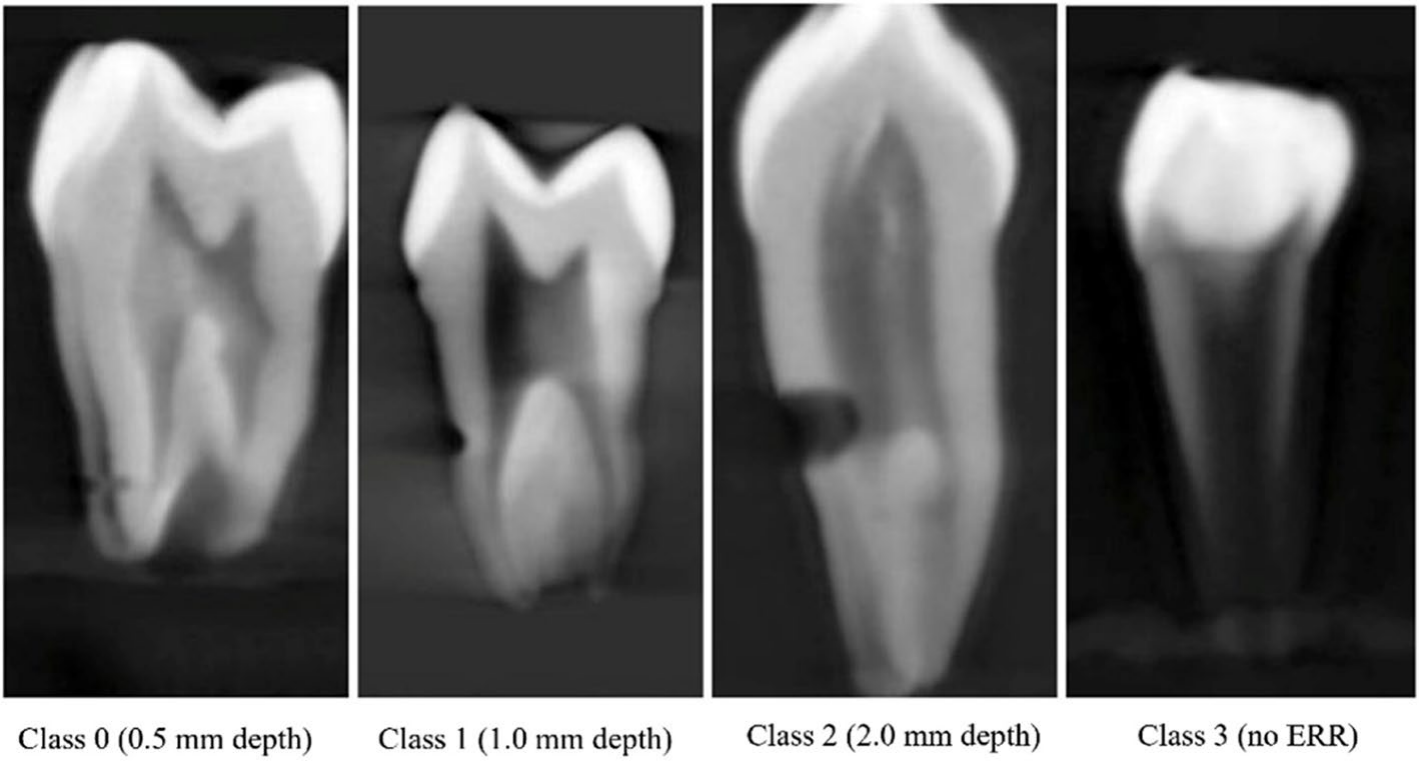
External root resorption sample images

Data analysis for ERR detection and labelling were performed by an oral and maxillofacial radiologist with five years of experience analyzing CBCT images and was considered as the ground truth. Each annotation was further classified into four groups of depths. All CBCT data was visualized on a Dell laptop (1920 × 1080 pixels, Dell Latitude E7450; Dell, Austin, TX). The ground truths dataset was prepared by segmenting the CBCT images (DICOM format) using a third-party A.I. tool (Makesense.AI) [32]. Teeth were grouped as 0 (ERR depth = 0.5 mm), 1 (ERR depth = 1.0 mm), 2 (ERR depth = 2.0 mm), 3 (no ERR). Figure 2 shows sample images from the dataset collected from the Faculty of Dentistry, Universiti Malaya.

### AI Network architecture and training

#### Image preprocessing

**Fig. 3:**
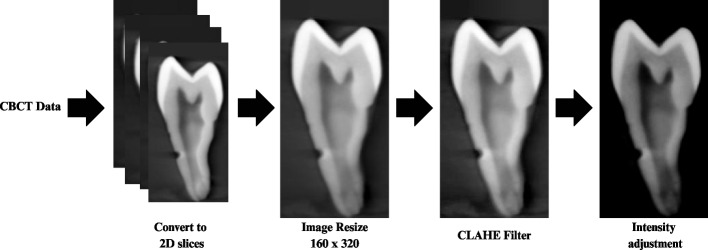
CBCT data image processing

In Phase 1, the extraction of region of interest (ROI) and image enhancement was performed (Fig. 3). A bounding box of 160 × 320 pixels was assigned to all 2D slices, with the tooth centered in the box and converted into Portable Network Graphic format. All sagittal slices were used to train and test (ROI) from these bounding boxes. The ROI obtained from a single tooth ranged from 17 to 80 slices resulting in a total of 2125 number of ROI extracted from the CBCT volumes (training and validation 1700, test 425). Then, the CLAHE filter was applied followed by an adjustment in image intensity before the pre-processing procedure.

#### Image classification

In Phase 2, four main DLMs (RF + VGG, RF + EFNET, SVM + VGG, and SVM + EFNET) were implemented to classify ERR lesions (Fig. 1). Subsequently, all four models were optimized using FST to produce four new enhanced DLMs (FS + RF + VGG, FS + RF + EFNET, FS + SVM + VGG and FS + SVM + EFNET). Training and testing ratios of 70:30 was selected as an optimum ratio for images classification as adopted in previous DLMs studies [33, 34]. Two-dimensional CBCT images of ERR were entered into a transfer learning with CNN models. In addition, these images were randomly distributed into training (70%), validation (10%) and test (20%) dataset. Subsequently, the ERR lesions observed in the images were classified as 0, 1,2 or 3 as the output, according to the depth of ERR in the images. In VGG16 and EfficientnetB4 systems, 555,328 and 18,764,579 parameters were utilized (Tables 1 and 2) [30]. Multiclass classification was performed by all models using Tensorflow and Keras phyton deep learning library.

**Table 1:** VGG16 parameters

Layer (Type)	Output shape	Parameter
Block1_conv1(Conv2D)	(None, 320, 160, 64)	1792
Block1_conv2(Conv2D)	(None, 320, 160, 64)	36,928
Block1_pool (MaxPooling2D)	(None, 160, 80, 64)	0
Block1_conv1(Conv2D)	(None, 160, 80, 128)	73,856
Block1_conv2(Conv2D)	(None, 160, 80, 128)	147,584
Block2_pool (MaxPooling2D)	(None, 80, 40, 128)	0
Block3_conv1 (Conv2D)	(None, 80, 40, 256)	295,168

Total parameters: 555,328

Trainable parameters: 555,328

Non-trainable parameters: 0

**Table 2:** EfficientnetB4 parameters

Layers (Type)	Output Shape	Parameters
EfficientnetB4 (Functional)	(None, 1792)	17,673,823
Table 2: EfficientNetB4 Parameters Module_wrapper_4	(None, 1792)	0
Module_wrapper_5	(None, 512)	918,016
Module_wrapper_6	(None, 256)	131,328
Module_wrapper_7	(None, 128)	32,896
Module_wrapper_8	(None, 64)	8256
Module_wrapper_9	(None, 4)	260

Total parameters: 18,764,579

Trainable parameters: 1,090,756

Non-trainable parameters: 17,673,823

#### Performance evaluation

The model’s performance was evaluated based on the calculation of accuracy. A confusion matrix summarized the prediction results on a classification task [35]. Five metrics were used to demonstrate the classification model’s performance: classification accuracy, F1-score, precision, specificity, and error rate [36]. Consequently, 70 values (7 × 10) were measured. The mean values for each group were calculated. Due to the non-normal distribution of the data, the Kruskal Wallis test, a non-parametric method, was employed to evaluate the difference in accuracy among all DLMs. The analysis was conducted using a statistical package for social sciences software (SPSS) 27.0 (IBM Corporation, Armonk, NY, USA). Following the Kruskal Wallis test for overall group differences, post-hoc analyses were conducted to examine pairwise differences between groups. Dunn’s post-hoc test was employed to identify specific pairs of groups with significant differences. Additionally, an independent *t*-test was conducted to assess any significant difference between the results obtained with and without FST. The metric evaluation was performed according to the following formula using confusion matrix in Table 3.

**Table 3:** Confusion matrix for binary classification

Data class	Classified as Positive	Classified as Negative
Positive	True Positive (TP)	False Negative (FN)
Negative	False Positive (FP)	True Negative (TN)

1 $$Accuracy = \frac{TP + TN}{TP + TN + FP + FN}$$

2 $$Specificity=\frac{ TN}{FP + TN}$$

3 $$Weighted\;accuracy=\frac1{\sum\nolimits_{i=1}^cw_i}\sum\nolimits_{i=1}^cW_i\;\mathrm X\;\left({\mathrm{TP}}_{\mathrm i}\;{\mathrm{xTN}}_{\mathrm i}\right)/\mathrm{Total}\;\mathrm{population}$$

4 $$F1 = \frac{2 TP}{2 TP + FP + FN}$$

5 $$Recall=\frac{ TP}{TP + FN}$$

6 $$Precision=\frac{Correct\;Detected\;ERR}{Correct\;Detected\;ERR+False\;Detected\;ERR}$$

7 $$Error\;Rate=\frac{\left(FP+FN\right)}{Total\;Population}$$

Where, TP = True Positive, FN = False Negative, TN = True Negative and FP = False Positive.

## Results

**Fig. 4:**
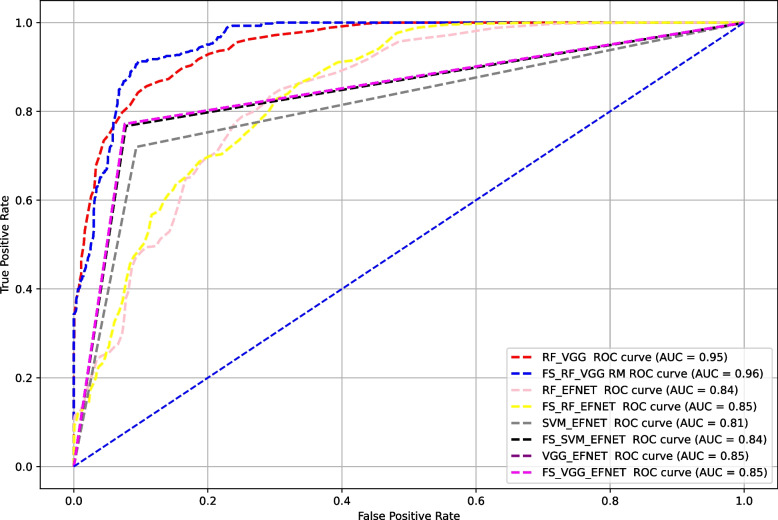
AUC of eight trained models

**Fig. 5:**
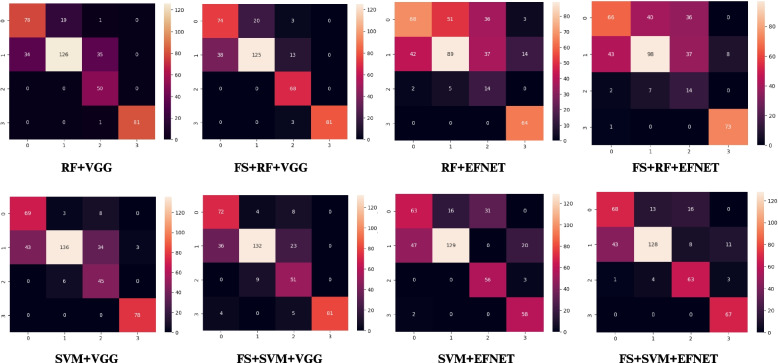
Confusion matrices showing prediction accuracy of all DLMS

The multiclass classification models’ performances were presented in Table 4. The highest performance was achieved by FS + RF + VGG model with overall accuracy of 81.9%, weighted accuracy of 83% and 81.9% F1-score, precision, and specificity. The error rate for FS + RF + VGG was 18%, and AUC of 96%. In contrast the lowest performing model was RF + EFNET with an overall accuracy of 55.3%, weighted accuracy of 61%, and 55.3% F1-score, precision, specificity. The error rate of RF + EFNET was 45%, and AUC of 84%. Following the implementation of FST, the highest accuracy improvement was achieved by SVM + EFNET model (4.7%) while the lowest improvement was recorded by the SVM + VGG model (1.7%). Of all eight DLMs, the highest AUC was recorded by FS + RF + VGG (96%), while the lowest was by SVM + VGG (81%) (Fig. 4). The Kruskal Wallis test showed a significant difference, with the *p*-value for the *H*-test being 0.008, which is less than the significant level at *α* = 0.05 (*p* < 0.05). This study indicated that there is a significant difference in accuracy between different models (*H* (7) = 19.119; *p* < 0.05) (Table 5). In Table 6, pairwise comparisons using Dunn’s post-hoc test among DLMs indicated a significant difference only between RF + EFNET and FS + RF + VGG (*p* < 0.05). Subsequent comparisons across other DLMs revealed no significant differences (*p* > 0.05). Furthermore, independent *t*-test showed no significant difference in the classification accuracy among allDLMs before and after incorporating FST (Table 7). The prediction accuracy of all eight DLMs were summarized in 4 × 4 confusion matrices as shown in Fig. 5.

**Table 4:** Comparison of classification performance between the deep learning-based systems

Models	Accuracy (Overall)	Accuracy (Weighted)	F1-score	Precision	Specificity	Error rate	AUC
RF + VGG	0.7882	0.82	0.7882	0.7882	0.7882	0.2117	0.95
FS + RF + VGG	0.8188	0.83	0.8188	0.8188	0.8188	0.18117	0.96
RF + EFNET	0.5529	0.61	0.5529	0.5529	0.5529	0.4471	0.84
FS + RF + EFNET	0.5906	0.61	0.5906	0.5906	0.5906	0.4094	0.85
SVM + VGG	0.7718	0.81	0.7718	0.7718	0.7718	0.2282	0.81
FS + SVM + VGG	0.7906	0.81	0.7906	0.7906	0.7906	0.2094	0.84
SVM + EFNET	0.7200	0.75	0.7200	0.7200	0.7200	0.2800	0.85
FS + SVM + EFNET	0.7671	0.79	0.7671	0.7671	0.7671	0.2329	0.85

**Table 5:** Kruskal Wallis test of significant difference in accuracy between different DLMs

Variable	Models	Mean Rank	Df	H	P (Sig)
Accuracy	RF + VGG	30.67	7	19.119	0.008*
	RF + EFNET	11.25			
	SVM + VGG	27.25			
	SVM + EFNET	18.50			
	FS + RF + VGG	37.83			
	FS + RF + EFNET	13.75			
	FS + SVM + VGG	32.92			
	FS + SVM + EFNET	23.83			

^*^Significant different *p* < 0.05

**Table 6:** Dunn’s post-hoc test between models

Sample 1- Sample 2	Test Statistic	Std. Error	Std. Test Statistic	Sig.	Adj. Sig. a
RF + EFNET-FS + RF + EFNET	-2.500	8.065	-0.310	0.757	1.000
RF + EFNET-SVM + EFNET	-7.250	8.065	-0.899	0.369	1.000
RF + EFNET-FS + SVM + EFNET	-12.583	8.065	-1.560	0.119	1.000
RF + EFNET-SVM + VGG	-16.000	8.065	-1.984	0.047	1.000
RF + EFNET-RF + VGG	19.417	8.065	2.408	0.016	0.450
RF + EFNET-FS + SVM + VGG	-21.667	8.065	-2.687	0.007	0.202
**RF + EFNET-FS + RF + VGG**	**-26.583**	**8.065**	**-3.296**	**0.001**	0.027*
FS + RF + EFNET-SVM + EFNET	4.750	8.065	0.589	0.556	1.000
FS + RF + EFNET-FS + SVM + EFNET	-10.083	8.065	-1.250	0.211	1.000
FS + RF + EFNET-SVM + VGG	13.500	8.065	1.674	0.094	1.000
FS + RF + EFNET-RF + VGG	16.917	8.065	2.098	0.036	1.000
FS + RF + EFNET-FS + SVM + VGG	-19.167	8.065	-2.377	0.017	0.489
FS + RF + EFNET-FS + RF + VGG	24.083	8.065	2.986	0.003	0.079
SVM + EFNET-FS + SVM + EFNET	-5.333	8.065	-0.661	0.508	1.000
SVM + EFNET-SVM + VGG	8.750	8.065	1.085	0.278	1.000
SVM + EFNET-RF + VGG	12.167	8.065	1.509	0.131	1.000
SVM + EFNET-FS + SVM + VGG	-14.417	8.065	-1.788	0.074	1.000
SVM + EFNET-FS + RF + VGG	-19.333	8.065	-2.397	0.017	0.463
FS + SVM + EFNET-SVM + VGG	3.417	8.065	0.424	0.672	1.000
FS + SVM + EFNET-RF + VGG	6.833	8.065	0.847	0.397	1.000
FS + SVM + EFNET-FS + SVM + VGG	9.083	8.065	1.126	0.26	1.000
FS + SVM + EFNET-FS + RF + VGG	14.000	8.065	1.736	0.083	1.000
SVM + VGG-RF + VGG	3.417	8.065	0.424	0.672	1.000
SVM + VGG-FS + SVM + VGG	-5.667	8.065	-0.703	0.482	1.000
SVM + VGG-FS + RF + VGG	-10.583	8.065	-1.312	0.189	1.000
RF + VGG-FS + SVM + VGG	-2.250	8.065	-0.279	0.78	1.000
RF + VGG-FS + RF + VGG	-7.167	8.065	-0.889	0.374	1.000
FS + SVM + VGG-FS + RF + VGG	4.917	8.065	0.610	0.542	1.000

Each row tests the null hypothesis that the Sample 1 and Sample 2 distributions are the same

Asymptotic significances (2-sided tests) are displayed. The significance level is 0.050

a Significance values have been adjusted by the Bonferroni correction for multiple tests

^*^Significant different *p* < 0.05

**Table 7:** Independent sample *t*-test of accuracy improvement with Boruta (FST algorithm)

Variable	FST	Mean	Standard Deviation	t	df	P (Sig)
Accuracy	No FST	0.77	0.107	-0.458	22	0.651
	With FST	0.79	0.102			

## Discussion

Precise detection of ERR lesions is crucial in preventing inaccurate management of this lesion which may subsequently result in irreversible root surface loss, discomfort, and non-vital tooth [1]. In this study, the performance of four DLMs on ERR identification were assessed using five parameters (classification accuracy, F1-score, precision, specificity, and AUC). Subsequently, the effect of FST on the DLMs performance was evaluated. The present study provides valuable insights into the potential of advanced machine learning techniques in improving ERR identification. To the best of our knowledge, the present study is the first to report the multiclass classification of ERR based on different depths of the lesions on CBCT images.

Deep learning-based algorithms play a significant role in developing an automated computer-aided diagnosis system for medical and dental radiographic image annotation, segmentation, and classification [19, 37–39]. Most deep learning algorithms require balance [40] and large data [41] to optimize an enormous number of weighting parameters in deep CNN. Hence, the current study introduced a transfer learning approach using pre-trained deep CNN algorithms to extract features from ERR lesions. Recent studies have reported that classification models incorporating pre-trained VGG16 and EfficientNetB4 architectures displayed robust performance in medical image analysis [19, 42]. The highest DLM accuracy of the current study was comparable to previous studies that had employed VGG16 for facial feature and jaw tumor classification [43, 44]. In the present study RF + EFNET demonstrated the lowest performance accuracy (0.55) than the other tested DLMs (RF + VGG, SVM + VGG, SVM + EFNET) i.e., more than 0.72. The performance of RF + EFNET in this study was even lower than previous ERR studies using panoramic radiograph [45, 46]. This can be attributed to a lack of compatibility between the RF classifier and the EfficientNetB4 algorithm used in this study. In general, DLMs had demonstrated a promising potential in assisting the identification and classification of ERR based on the lesion’s depth.

Feature selection technique (FST) can improve classification model performance by identifying and selecting the most informative features within the dataset [29, 47]. The utilization of FST, especially Burota and RFE, had decreased the risks associated with overfitting and improved the interpretability of medical image analysis [23, 48, 49]. The present study observed an increase in DLMs accuracy improvements (2–4.7%) when FST were combined during the post-processing phase. Similarly, high accuracy improvements (10% and 5.8%) were reported by previous studies using Burota [50] and RFE [29]. The low accuracy improvement that was observed in the current study might be due to imbalanced classes of the dataset, with a greater amount of data in 0.5 and 1.0 mm classes [51]. All DLMs in this study demonstrated improvement in classification accuracy. A study using FST on neurodegenerative lesions classification reported a selective DLM accuracy improvement (CfsSubsetEval, WrapperSubsetEval, ChiSquaredAttributeEval, and ClassifierAttributeEval) [52]. Therefore, it can be assumed that accuracy improvement might be influenced by the compatibility of FST and hybrid DLMs utilized in this study, as reported by Bhalaji et al. and Albashish et al. [53, 54].

In this present study, DLM systems have demonstrated considerable performance in identifying ERR. The main limitation was identified during conducting this study, namely the small CBCT dataset. To avoid overfitting due to small sample size, this study had utilized a high-quality training dataset specifically to emphasize ERR depths [55]. Furthermore, data augmentation [56] was performed to increase the training dataset, and transfer learning approach (VGG16 and EfficienNetB4) was implemented to enhance the performance of DLMs [57]. This study had exclusively utilized DLMs in identifying ERR on extracted premolar teeth. However, the ability of these newly developed models should be tested on real data before clinical applications. Although the experimental nature of this study might compromise the ability of these DLMs on real data [58], it allows standardized preparation techniques for ERR and CBCT scanning parameters [59]. Future research should focus on three main areas: expanding the dataset, exploring the ability of various FSTs, and conducting prospective clinical trials.

## Conclusions

The present study explored the potential of eight newly developed DLMs in identifying ERR on CBCT images. The application of deep learning-based algorithms on CBCT images had demonstrated promising results for future automated ERR identification. Integrating compatible FST with deep learning-based models may enhance the performance of all DLMs in identifying ERR lesions.

## Supplementary Information

**Additional file 1.**

## Data Availability

The datasets used and analysed during the current study are available from the corresponding author on reasonable request.
