# Correction: Application of deep learning and feature selection technique on external root resorption identification on CBCT images

**DOI:** 10.1186/s12903-024-05030-x

**Published:** 2025-01-31

**Authors:** Nor Hidayah Reduwan, Azwatee Abdul Aziz, Roziana Mohd Razi, Erma Rahayu Mohd Faizal Abdullah, Seyed Matin Mazloom Nezhad, Meghna Gohain, Norliza Ibrahim

**Affiliations:** 1 Department of Oral and Maxillofacial Clinical Sciences, Faculty of Dentistry, Universiti Malaya, Kuala Lumpur, 50603 Malaysia; 2 Department of Restorative Dentistry, Faculty of Dentistry, Universiti Malaya, Kuala Lumpur, 50603 Malaysia; 3 Department of Pediatric Dentistry and Orthodontic, Faculty of Dentistry, Universiti Malaya, Kuala Lumpur, 50603 Malaysia; 4 Department of Artificial Intelligence, Faculty of Computer Science and Information Technology, Universiti Malaya, Kuala Lumpur, 50603 Malaysia; 5 Centre of Oral and Maxillofacial Diagnostic and Medicine Studies, Faculty of Dentistry, University Teknologi MARA, Sungai Buloh, 47000 Malaysia

**Correction to: BMC Oral Health (2024) 24:252**

10.1186/s12903-024-03910-w

In this article [1], the author’s name Azwatee Abdul Aziz was incorrectly written as Azwatee Abdul Abdul Aziz.

The original article has been corrected.
