# Supplementary material for: Application of deep learning and feature selection technique on external root resorption identification on CBCT images
Source: BMC Oral Health. 2024 Feb 19;24:252. doi: 10.1186/s12903-024-03910-w (PMC10875886; doi:10.1186/s12903-024-03910-w)
Supplement: Supplementary file 1 — Additional file 1. [file 12903_2024_3910_MOESM1_ESM.docx]

Supplementary materials

1. Image Dataset

<https://drive.google.com/drive/folders/1-NDp-3Sh6CrWk_WOeHfv4AqEgqccGiCu?usp=drive_link>

1. Ground Truth Labelling

<https://drive.google.com/drive/folders/1Wx4ysKEsd5jEab3Q0_aTAT-wjxrFdtFr?usp=drive_link>
